# Correction to: An analysis of the kinetics of molecular response during the first trimester of treatment with nilotinib in newly diagnosed chronic myeloid leukemia patients in chronic phase

**DOI:** 10.1007/s00432-017-2532-1

**Published:** 2017-10-23

**Authors:** Juan Luis Steegmann, Dolors Colomer, Maria-Teresa Gómez-Casares, Valentín García-Gutiérrez, Guillermo Ortí, Angel Ramírez-Payer, Eduardo Olavarria, Ferrán Vall-llovera, Pilar Giraldo, Eulogio Conde, Rolando Vallansot, Jose Luis López-Lorenzo, Luis Palomera, Alberto Álvarez-Larrán, Venancio Conesa, Guiomar Bautista, Laura Casas, Frank Giles, Andreas Hochhaus, Luis Felipe Casado-Montero

**Affiliations:** 1 Hematology Service, Advanced Therapies in Oncohematology, IIS-IP, Hospital de la Princesa, Madrid, Spain; 2 Unitat d’ Hematopatologia, Hospital Clinic, Barcelona, Spain; 3 Laboratorio de Hematología, Biología Molecular, Hospital Universitario de Gran Canaria Dr.Negrín, Las Palmas, Spain; 4 Servicio de Hematología, Hospital Universitario Ramón y Cajal, IRYCIS, Madrid, Spain; 5 Hematology Department, Hospital Universitari Vall d’Hebron, VHIO, Barcelona, Spain; 6 Servicio de Hematología, Hospital Universitario Central de Asturias, Oviedo, Spain; 7 Hematology Department, Imperial College Healthcare NHS Trust, London, UK; 8 Servicio de Hematología, Hospital Universitari Mútua Terrassa, Terrassa, Spain; 9 Instituto Investigación Sanitaria Aragón (IIS Aragon), CIBERER, Zaragoza, Spain; 10 Hematologia, Universidad de Cantabria, Santander, Spain; 11 Servei d’Hematologia, ICO-Tarragona, Hospital Universitari Joan XXIII, Tarragona, Spain; 12 Hematologia, Clínica de la Concepción, Madrid, Spain; 13 Hospital Clínico Universitario Lozano Blesa, Instituto Investigación (ISS) Aragón, Zaragoza, Spain; 14 Hematology Department, Hospital del Mar-IMIM, Barcelona, Spain; 15 Hematología, Departament de Salut d’Elx, Hospital General, Elche, Spain; 16 Hematología, Hospital Puerta de Hierro, Madrid, Spain; 17 Statistics Department, Dynamic Solutions, SL, Barcelona, Spain; 18 Division of Hematology Oncology, Northwestern University Feinberg School of Medicine, Chicago, USA; 19 Klinik für Innere Medizin II. Hämatologie/Onkologie, Universitätsklinikum Jena, Jena, Germany; 20 Servicio de Hematología y Hemoterapia, Hospital Virgen de la Salud, Toledo, Spain

## Correction to: J Cancer Res Clin Oncol (2017) 143:2059–2066 DOI 10.1007/s00432-017-2445-z

The article “An analysis of the kinetics of molecular response during the first trimester of treatment with nilotinib in newly diagnosed chronic myeloid leukemia patients in chronic phase”, written by Juan Luis Steegmann, Dolors Colomer, Maria-Teresa Gómez-Casares, Valentín García-Gutiérrez, Guillermo Ortí, Angel Ramírez-Payer, Eduardo Olavarria, Ferrán Vall-llovera, Pilar Giraldo, Eulogio Conde, Rolando Vallansot, Jose Luis López-Lorenzo, Luis Palomera, Alberto Álvarez-Larrán, Venancio Conesa, Guiomar Bautista, Laura Casas, Frank Giles, Andreas Hochhaus, Luis Felipe Casado-Montero, was originally published Online First without open access.

After publication in volume 143, issue 10, pages 2059–2066 the authors decided to opt for Open Choice and to make the article an open access publication. Therefore, the copyright of the article has been changed to © The Author(s) 2017 and the article is forthwith distributed under the terms of the Creative Commons Attribution 4.0 International License (http://creativecommons.org/licenses/by/4.0/), which permits use, duplication, adaptation, distribution and reproduction in any medium or format, as long as you give appropriate credit to the original author(s) and the source, provide a link to the Creative Commons license, and indicate if changes were made.

